# Vancouver’s Alcohol Knowledge Exchange: lessons learned from creating a peer-involved alcohol harm reduction strategy in Vancouver’s Downtown Eastside

**DOI:** 10.1186/s12954-023-00838-2

**Published:** 2023-07-26

**Authors:** Aaron Bailey, Brittany Graham, Myles Harps, George Sedore

**Affiliations:** Eastside Illicit Drinkers Group for Education, Vancouver Area Network of Drug Users, 380 East Hastings Street, Vancouver, BC V6A 1P4 Canada

**Keywords:** Illicit drinking, Alcohol harm reduction, Community engagement, Alcohol policy, Downtown Eastside, Vancouver Alcohol Strategy, Alcohol Knowledge Exchange, EIDGE, VANDU

## Abstract

Despite high rates of harm attributable to alcohol use itself and the associated marginalization of illicit drinkers in Vancouver’s Downtown Eastside (DTES), alcohol-specific harm reduction services there are under-resourced and highly disconnected from one another. In response to these conditions and high rates of death amongst its membership, the Eastside Illicit Drinkers Group for Education, an affiliate group of the Vancouver Area Network of Drug Users, convened a regular meeting of stakeholders, termed a “community of practice” in 2019 to bring together peers who used beverage and non-beverage alcohol, shelter and harm reduction service providers, public health professionals, clinicians, and policymakers to improve system-level capacity to reduce alcohol-related harm. The discussions that followed from these meetings were transformed into the Vancouver Alcohol Strategy (VAS), a comprehensive, harm reduction-oriented policy framework for alcohol harm reduction in the DTES. This article highlights our experiences producing community-led alcohol policy through the VAS with specific attention to the ways in which people who use alcohol themselves were centred throughout the policy development process. We also provide summary overviews of each of the VAS document’s 6 thematic areas for action, highlighting a sampling of the 47 total unique recommendations. Historically, people who use non-beverage alcohol and whose use of alcohol in public spaces is criminalized due to housing precarity and visible poverty have been excluded from the development of population-level alcohol policies that can harm this specific population. The process of policy development undertaken by the VAS has attempted to resist this top-down approach to public health policy development related to alcohol control by intentionally creating space for people with lived experience to guide our recommendations. We conclude by suggesting that a grassroots enthusiasm for harm reduction focused policy development exists in Vancouver’s DTES, and requires resources from governmental public health institutions to meaningfully prevent and reduce alcohol-related and policy-induced harms.

## Introduction and background

Drug poisoning deaths in Vancouver reached an all-time high in 2021 in the absence of a sufficiently accessible safe supply of opioids and stimulants. The continued adulteration of the illicit drug supply with fentanyl, fentanyl analogues, and higher concentrations of benzodiazepines was a leading contributor to 2224 deaths in 2021, representing the highest recorded total since the declaration of a public health crisis by the provincial government in 2016 [1]. People who use beverage and non-beverage alcohol in addition to unregulated opioids face heightened risk of fentanyl-related drug poisoning death, evidenced by alcohol’s previously recorded involvement in approximately 1/3rd of suspected drug poisoning deaths in Vancouver [2]. Alcohol-specific harm reduction services remain problematically absent or fragmented throughout much of the city and province. This service gap is especially acute in Vancouver’s Downtown Eastside (DTES), where illicit drinkers experience disproportionately high-rates of alcohol-related harm and polydrug poisoning-related death [3–8]. These outcomes are deeply connected to the longstanding exclusion of people who use illicit alcohol from the harm reduction movement and broader forms of structural marginalization related to the ongoing violence of settler colonialism in Canada, legislated poverty, systemic racism, stigmatizing organization of health service delivery, and the financialization of housing [4, 6].

In this commentary, we (AB, BG, MH, GS, and EIDGE) reflect on how we, writing from the perspective of program staff (AB, BG) advised closely by illicit drinkers (MH, GS, EIDGE), resisted illicit drinkers longstanding disconnection from alcohol policy development in our approach to collaboratively producing a novel, community-directed vision for alcohol harm reduction in Vancouver. To do this, we convened the Alcohol Knowledge Exchange (AKE), a British Columbia-centred series regular meeting of individuals and organizations working in the alcohol-specific health services and policy sectors, termed a “community of practice”, to encourage intersectoral collaboration between service providers and people with lived experience of illicit alcohol use and intersecting structural marginalization. We then worked with peers to write the Vancouver Alcohol Strategy (VAS), a comprehensive plan for alcohol policy reform presented to Vancouver Coastal Health (VCH). By bringing the alcohol policy development process to illicit drinkers themselves, namely, the Eastside Illicit Drinkers Group for Education (EIDGE) and PHS Drinkers Lounge Community Managed Alcohol Program (aka The Drinkers Lounge) effectively resisted the exclusionary and highly professionalised sphere of alcohol policy design and implementation. We conclude by offering key lessons for equity-focused alcohol policy development for public health policymakers and practitioners, taking care to emphasise the importance of respecting and creating space for the lived experience of peers who use remains use illicit alcohol.

The historical, colonial, and political context of Vancouver’s DTES is integral to understanding the VAS’ place within it. Established through the non-consensual colonial dispossession of Musqueam, Squamish, and Tsleil-Waututh lands, the DTES was Vancouver’s first downtown core [9, 10]. The intersection of Main and Hastings Streets is the geographic center of the area, which includes other popular landmarks including Oppenheimer Park and the Carnegie Community Centre. The neighbourhood includes a unique concentration of Single Room Occupancy hotels (SROs), notoriously poorly maintained former short stay hotels with shared bathrooms and kitchen facilities [11]. Non-profit managed and private SROs serve as the majority of the city’s housing for extremely low-income people [11–13]. Public health’s relationship with the neighbourhood is historically fraught. Japanese and Chinese–Canadians, precariously housed drinkers, Indigenous residents, and people who use drugs, and SRO tenants throughout the DTES and Vancouver’s adjacent Chinatown have historically been approached by institutions of public health as sources of disorder and contamination. Prior to the second world war, the substandard living conditions of Chinese–Canadians, a result of racist planning practices and landlord–tenant relations, resulted in the routine condemnation of dwellings [11, 14, 15]. A different arm of the Canadian state forced Japanese–Canadian residents, including a large community of SRO caretakers, into detention camps and expropriated familial property beginning in 1941 [16, 17]. The 1950s saw local public health authorities focus intensively on the professionalized management of the “Skid Road” drinker, typified as an elderly, male ex-resource worker whose chronic health conditions and unsightly appearance weighed down the public purse and nearby property values [18]. Eventually, collaboration between civic politicians, health officials, and neighbourhood advocates would lead to the closure of the areas liquor store and the passage of a moratorium on liquor licenses, both of which were associated with increased non-beverage alcohol use in the neighbourhood [18].

Between 1970 and the early 1990s, the continuing cultural and geographic dislocation of Indigenous people, the unsupported shuttering of psychiatric institutions, worsening volatility of the unregulated drug market, stagnant income assistance rates, rising income inequality, arrival of HIV/AIDS, police-led kettling of vulnerable sex workers into the DTES, spiking Hepatitis C incidence, repeat overdose crises, public health’s departure from housing, and federal divestment from social housing resulted in intensified health intervention in the neighbourhood [9, 10, 17, 19–22]. These conditions prompted the creation of drug user-led advocacy and harm reduction service providing organizations, many of whom used civil disobedience to implement what have since become accepted best practices in the prevention of blood borne disease transmission, overdose prevention, and community empowerment [19, 20, 23–25]. Successful challenges to municipal moratoriums on harm reduction funding and a Supreme Court of Canada permitting North Americas first Supervised Injection Facility, InSite, to operate legally in 2011, naloxone distribution, and the creation of unsanctioned safe supply compassion clubs are notable example of this form activism [23–26]. The health status of DTES residents has been historically over researched, prompting many organizations in the community to develop independent ethical standards and guidelines for participation in potentially exploitative work. Since 2015, the DTES has experienced extremely high rates of illicit drug poisoning and related death as the number of unsheltered residents rises with the loss of affordable SRO units [27–29]. Extremely high rates of death amongst people who use illicit drugs have once again driven the community to conceive of new policy solutions to protect those most at risk of drug poisoning, including illicit drinkers.

**Fig. 1:**
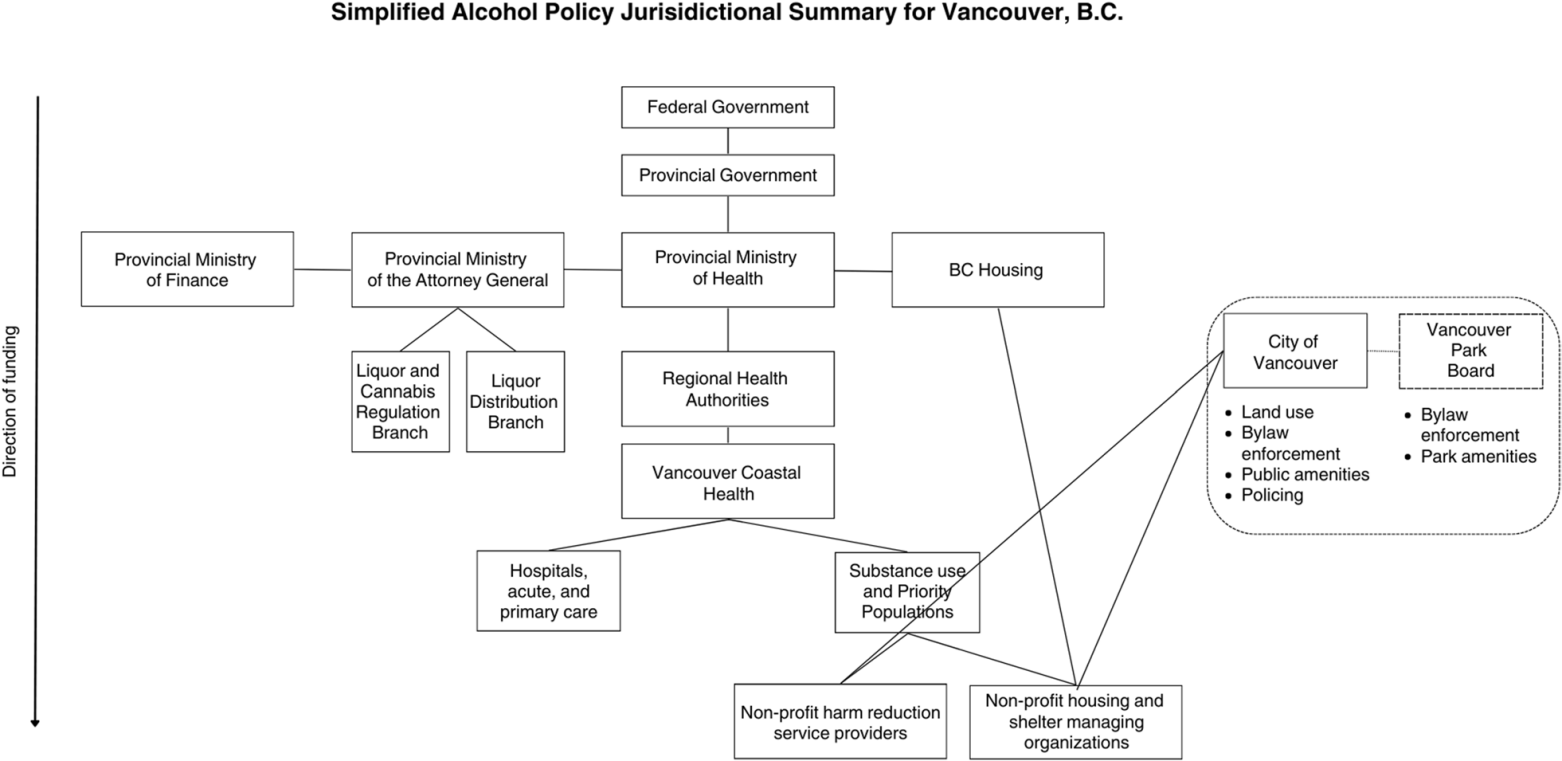
Responsibility for alcohol policy in Vancouver falls to several levels of government, making coordinated action to address alcohol-related harms difficult for drug user groups

Responsibility for creating and implementing alcohol related health policies falls to different levels of government in Vancouver. Health Canada is responsible for the regulation of some alcohol taxation and industry regulation. The Province of B.C. is responsible for the creation and administration of liquor retail systems, whole sale purchasing for public monopolies, issuing liquor licenses, enforcing liquor laws including minimum purchase ages through the Ministry of the Attorney General’s Liquor and Cannabis Regulation and Liquor Distribution Branches. B.C.’s Ministry of Finance collects and distributes alcohol excise taxes, while the Ministry of Health finances the delivery of a single-payer acute care health system. Beginning in the late 1980s, delivery of local public health services (and clinical care) in Vancouver was transferred from the former Vancouver Health Department to a collection of Regional Health Authorities under the direction of the Provincial Ministry of Health, most prominently Vancouver Coastal Health (VCH). The latter organization plays the most direct role in allocating funds for public health and clinical care in Vancouver. At the local level, City of Vancouver and Vancouver Parks Board are two legally distinct entities that play key roles in the creation and enforcement of alcohol policy. The City of Vancouver is responsible for determining, where liquor outlets can be located, the enforcement of bylaws including those prohibiting public consumption of alcohol, and the regulation of police department. Similarly, the Vancouver Park Board oversees the enforcement of bylaws related to alcohol use in parks. Figure 1 depicts an unofficial summary of the organization of public health policy jurisdictions as they relate to alcohol and touch down in Vancouver's DTES.

Neoliberal health governance looms large over the structure of alcohol-related policy and service delivery in the DTES. The creation of the Regional Health Authority system in B.C. reflected a national trend towards regionalization and governmental downloading of health services across Canada beginning in the 1990s [30, 31]. Since this time, non-profit service providing organizations who receive funding from Regional Health Authorities have played a central role in the direct delivery of harm reduction and other social services, housing and shelter, health services, and supportive housing. While a large number of non-profit organizations operating in the DTES are well-funded, they can be viewed as filling highly visible gaps in Canada’s declining welfare state [32]. The organization with which EIDGE is affiliated, the Vancouver Area Network of Drug Users (VANDU), is an example of a member-governed non-profit organization that has served current and former illicit drug users since 1997 [19, 20, 23–25].

## Illicit drinking and alcohol-related harm in the DTES

The AKE project and writing of the VAS originated as an initiative of EIDGE, a peer-led education and advocacy group for illicit drinkers operated under the umbrella of VANDU. Illicit drinkers are people who drink alcohol substitutes not meant for human consumption (e.g., rubbing alcohol, mouthwash, and illegally produced homemade alcohol), and/or who drink beverage alcohol (e.g., beer and wine) in a way that is criminalized, like in unsanctioned public spaces [6, 7]. Illicit drinkers’ use of public space is a result of visible poverty, namely, housing precarity, the gradual worsening of Vancouver’s housing crisis, the commonality of residing in Single Room Occupancy hotels (SROs) without common areas for socialising, intensive policing, and closure of neighbourhood parks. The closure of neighbourhood pubs serving affordable alcohol and the few community spaces that accommodate drinkers throughout the neighbourhood during the COVID-19 pandemic intensified this coercion. The membership of EIDGE is primarily composed of people with long-term and heavy patterns of alcohol consumption who experience intense structural marginalization. We use the latter phrase to refer to members’ typical experience of concurrent, intersecting historical and political forces that disempower, harm, and otherwise oppress. For example, it is common for EIDGE members to have experienced extended episodes of homelessness, familial abuse, intergenerational trauma, discrimination in health care settings, interaction with the Indigenous child welfare system, incarceration, adverse childhood experiences, and familial experiences in Canada’s genocidal residential school system. Many members have also repeatedly faced policy-based barriers to well-being embedded within B.C.’s system of addictions care. Smoking bans and long waits at detox sites, a lack of detox sites capable of supporting alcohol and benzodiazepine withdrawals, an insufficient number of in-patient treatment beds, a largely unregulated treatment sector, and a lack of post-discharge recovery prevent long-term, heavy drinkers from accessing said services. To avoid dangerously acute alcohol withdrawal symptoms, drinkers in the DTES often choose to consume harmful non-beverage alcohol substitutes, because they are cheaper and more geographically accessible than affordable beverage alcohol in the neighbourhood [7, 8]. Drinkers’ structural marginalization is reflected by extremely high risk of drug poisoning death, emergency department use, police harassment, adulterant poisoning, hepatic injury, experience of interpersonal violence, robbery, accidents, hypothermia, dehydration, and traffic-related injury amongst our membership [6–8]. The ongoing violence of settler colonialism is a key driver of these and other alcohol-related harms for our membership, 80% of whom identify as Indigenous [8]. 

## Vancouver’s alcohol harm reduction landscape

These disturbingly high rates of alcohol and other drug-related harm amongst our membership have not been accompanied by seemingly appropriate policy responses from Provincial and regional public health authorities. Alcohol has remained largely an afterthought within a harm reduction sphere that has been, understandably, focused largely on opioids and stimulant drugs for decades. Despite a commitment to scale up evidence-based Managed Alcohol Programs (MAPs) in VCH’s Second Generation Strategy, the demand for MAP services continues to outpace the capacity of the Drinkers Lounge, which is the only MAP accepting clients identified in the DTES [33, 34]. Over the last 12 years of the group’s operation, EIDGE members (MH, GS) and program staff (AB, BG) have informally documented a number of repeat systemic concerns shared by the illicit drinking community. We recount those experiences together here. Drinkers anecdotally report that programming spaces including shelters, community centres, and clinics throughout the DTES are not equipped or willing to accommodate drinkers, who are often turned away or prohibited from using alcohol in any form while accessing services. For drinkers who wish to access alcohol-specific detox or in-patient recovery, beds-on-demand are usually not available, leaving service-users without follow-up support when leaving these programs. Smoking bans in detox facilities reportedly create a hostile environment for drinkers and people who use other drugs, and home detox programs are scarcely resourced or promoted as this population are viewed as high risk and do not fit the criteria of programs, such as VCH’s START team. Many EIDGE members have also cited hospital-specific barriers to receiving proper care, including shortages of gold standard medications including Acamprosate, dismissal of alcohol withdrawal symptoms by medical staff and difficulty accessing in-hospital MAP services. Meanwhile, the frequent removal of public amenities including benches and bus stops, and the daily confiscation of makeshift shelters on East Hastings St by the City of Vancouver and Vancouver Park Board has continued to criminalize and displace illicit drinkers. These conditions are not entirely unique to DTES, but in our collective experience as illicit drinkers and supporting staff persons, they are important determinants of drinkers well-being in this neighbourhood.

Alcohol has been a priority of population-level public health policy in British Columbia. Across B.C, forms of alcohol control that emphasise supply reduction, including provincial excise taxes and local restrictions on the availability of beverage alcohol, are an evidence-based means of reducing alcohol-related harm for a significant segment of the general population [35–41]. However, in the unique context of illicit drinking in the DTES, these efforts to make beverage alcohol less accessible are significant drivers of non-beverage alcohol consumption and have a highly regressive impact on drinkers living with severe alcohol dependency and extremely low incomes [42, 43]. For example, people who use non-beverage alcohol frequently state that their reasons for doing so are related to its low price and ease of accessibility relative to beverage alcohol [7, 44]. When faced with the possibility of severe withdrawal symptoms low-income drinkers will choose to drink a non-beverage alcohol to mitigate harms from possible withdrawal, including seizures, dangerous cardiovascular effects, and delirium [45]. EIDGE and the Drinkers Lounge have articulated a different approach for the DTES that balances evidence-based population-level alcohol control science with a pragmatic and equity-oriented form of alcohol harm reduction.

In early 2020, Vancouver’s alcohol-specific health service and harm reduction landscape was described by illicit drinkers, VANDU staff, and the management of the PHS Drinkers Lounge and clinicians as siloed, fragmented, and ineffective at meeting people with long term, heavy drinking patterns in the DTES, where they are. This apparent lack of system-level capacity was made more visible by the onset of the COVID-19 pandemic, when it became nearly impossible for frontline staff at these organizations to determine which alcohol-specific programs had continued to operate and how. In our experience as supporting staff at VANDU (AB, BG) often tasked with system navigation and members of EIDGE who have independently sought out alcohol-specific services (MH, GS), drinkers, service providers, medical professionals and policymakers did not have a clear understanding of the breadth of alcohol-related programming in the community or have the capacity to navigate the under-resourced service landscape. Governmental and non-profit organisations operating in the DTES lacked point-people on alcohol-related issues and did not know where to refer service-users to access information about alcohol harm reduction, detox, and in-patient treatment. Despite the existence of independently operating low-barrier programs and groups, a lack of communication and referral infrastructure has severely impaired the public health systems ability to reduce extreme alcohol-related harms in Vancouver. These conditions placed drinkers at the intersection of overlapping health emergencies. In addition to experiencing extremely high risk of acute and chronic harm related to alcohol use and the criminalization of illicit drinking, EIDGE members experienced a high number of drug poisoning deaths relative to other groups within VANDU since 2015. EIDGE and the Drinkers Lounge decided to act to address this siloing of services and personnel working with drinkers by bringing people who used illicit alcohol, policymakers, service providers, and medical professionals together for the first Alcohol Knowledge Exchange (AKE).

## The Alcohol Knowledge Exchange project

EIDGE identified the need for a convening process and sought capacity building funding from the City of Vancouver in 2019 to design and lead a project to address this system-level fragmentation. The City of Vancouver eventually deemed this request incompatible with the capacity building grant stream but referred the application to VCH. Soon after, VCH awarded EIDGE a one-time-only grant for a total of $25,520 in the winter of 2019. The work was organized around a series of official objectives: first, we would convene a community of practice and facilitate a series of meetings. We hoped that the physical bringing together of individuals and organisations working in Vancouver’s harm reduction sector and supporting clients who used alcohol would prompt more serious collaboration and mutual understanding of our city's service landscape. Second, the project was also to be organized around peer consultation, ensuring that illicit drinkers themselves were heard through the entire AKE project and responsible for directing its outputs whenever possible. Finally, we would translate the findings of the AKE project and peer insights into a unified vision for harm reduction-informed alcohol policy in Vancouver.

Before the planned in-person events of the AKE could commence, our timeline and project plan were significantly altered by the COVID-19 pandemic. It became clear that our original plan to convene in person in Vancouver, share food, and develop in-person relationships between AKE participants could not proceed. We quickly transitioned our planned programming to an online format, with the initial series of AKE meetings taking place over Zoom. This significantly hampered initial peer involvement in AKE work, which we will discuss later in this section. These online meetings were not, however, the beginning of the AKE project in earnest. Discussions with drinkers dating well before the 2019 funding decision were the backbone of the project and had previously revealed the degree of fragmentation that existed within Vancouver’s system of alcohol treatment, harm reduction, and care. Tentative priority areas for policy intervention for drinkers, based on years of meetings and individual conversations, were compiled by program staff (BG). These themes became the framework for early, Zoom-based AKE discussion in which only one or two peers could participate. These policy domains included a lack of available detox beds and Managed Alcohol Program capacity in the DTES, smoking bans in in-patient treatment and detox centres, and the ongoing removal and benches and other public amenities from their neighbourhood. Having established drinkers’ priorities throughout the weeks and months that preceded that initial meetings of the AKE community of practice, we began to host regular online meetings to which a network of 93 individual and organizational participants identified by VANDU and PHS Drinkers Lounge staff as being engaged in service provision or policy work related to alcohol harm reduction were regularly invited. While a smaller group of between 10 and 20 people typically attended each of the 6 AKE Zoom calls, all 93 participants received regular updates and invited to contribute to the eventual recommendations of the VAS. Both groups consisted of health care professionals, non-profit leadership, people with lived and living experience of alcohol or substance use, frontline service providers, ministerial staff, policymakers affiliated with VCH, representatives of other regional health authorities, and the City of Vancouver. Table 1 includes a detailed summary of each organization that was invited to form the Zoom and email-based AKE Community of Practice and the sectors they represented.

**Table 1:** Participants in the email and Zoom-based AKE community of practice

Organizations included in the AKE community of practice	Number of participants
Vancouver Coastal Health	24
PHS Community Services Society	13
Non-profit Housing Providers	7
British Columbia Centre for Substance Use	6
Indigenous health and social services organizations	5
Providence Health Care	4
British Columbia Centre for Disease Control	4
Academic researchers	4
First Nations Health Authority	4
Legal services	4
Detox and substance use treatment services	4
Independent practitioner	3
Vancouver General Hospital	2
British Columbia Ministry of Health	2
Youth-serving non-profit organizations	2
Person with lived and living experience	1
VANDU Staff	1
City of Vancouver	1
Community Action Initiative	1
British Columbia centre for Excellence in HIV/AIDS	1

**Fig. 2:**
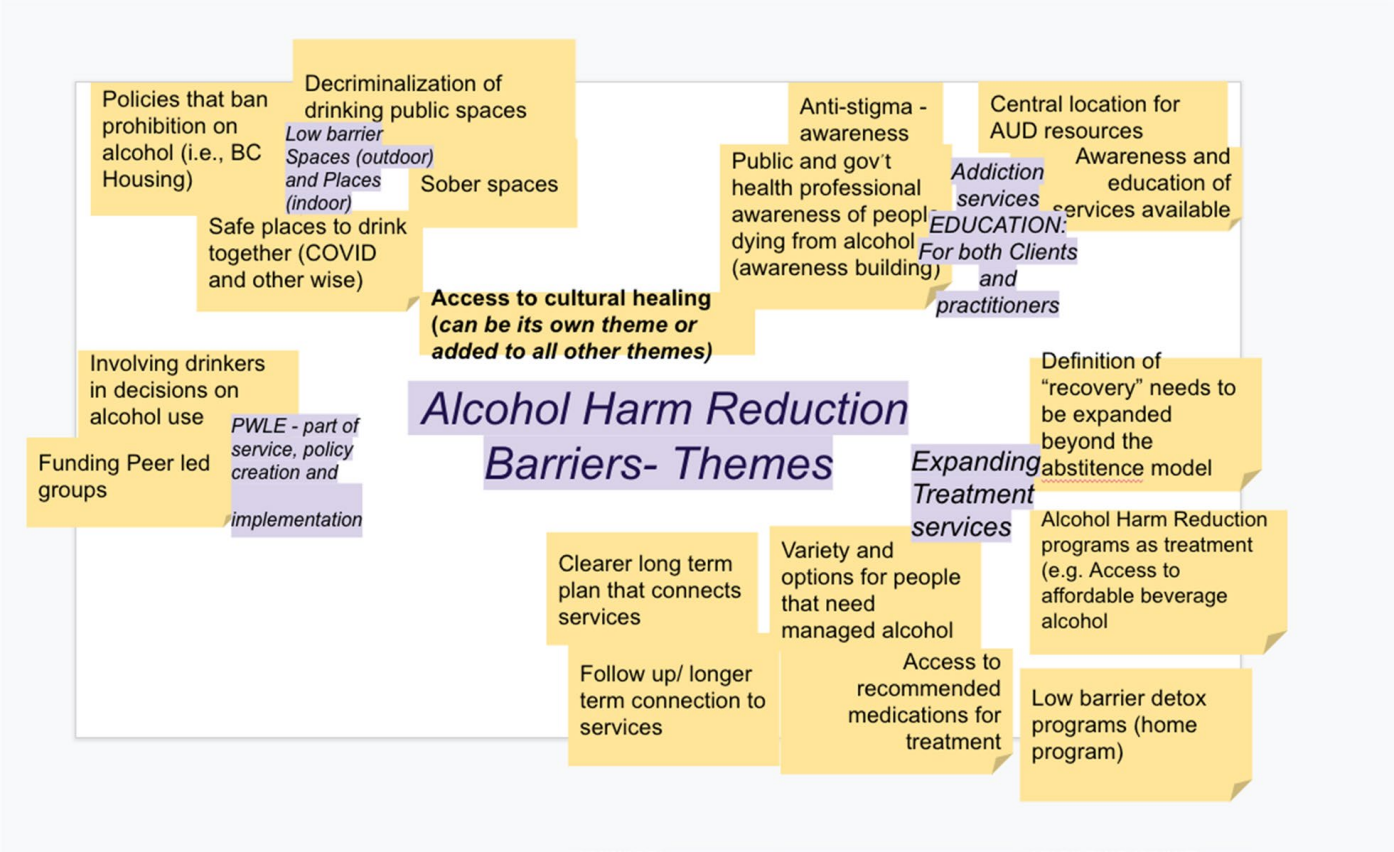
Thematic areas that would become the VAS’ Action Items come together through online discussion and support from a professional facilitator

Small samples of this group convened on Zoom 6 times between June of 2020 and March of 2021. During this time, 1–2 peers from EIDGE attended discussions supported by program staff. Received. Absent community of practice members also received regular email updates of AKE activities. We reviewed previous EIDGE research to discuss the state of illicit drinkers’ health and well-being, learned about the history of alcohol policy and harm reduction in the DTES, and began to categorise the main areas for policy action with the help of a professional facilitator. Several AKE meetings, with the help of this facilitator, focused on carrying out an informal, collaborative thematic sorting of the priority policy issues that had been raised to that point. In March of 2021, AB reviewed notes from AKE meetings, which included a thematic grouping and sorting activity (Fig. 2) and began work on an early draft of the VAS itself. This draft was a synthesis of conversations with drinkers prior to COVID-19 pandemic and a summary of the conversations of the AKE community of practice meetings grouped together into “Action Items”. Figure 2 depicts an early draft of these “Action Items” as they were drawn from AKE discussions by the facilitator. Importantly, the AKE process and the translation of these discussions into the VAS did not rely on formal qualitative methods or thematic analysis. We did not recruit participants according to specific criteria, transcribe sessions, and draw out our themes through deductive coding. Beyond the occasional support of a facilitator and consistent notetaking, informal discussion structured by participant’s experiences navigating alcohol-related services in Vancouver, referring illicit drinkers through said services, or creating policy was the primary source of “data”. This flexibility was intended to be pragmatic—EIDGE—and the PHS Drinkers Lounge sought to convene a group of people with combined decades of experience in alcohol policy and service delivery, determine common challenges and frustrations, agree on system-level demands for change, and document those demands. Assembling the AKE was also highly reliant on relationships built by frontline participants over years of working together in different ways. In this sense, the AKE and VAS are more accurately viewed as products of organic community organizing to create policy change more than they are examples of methodologically rigorous qualitative research. The latter framing, in this instance, was simply not seen as appropriate for the advocacy work prioritized by EIDGE in the contemporary moment. We discuss this distinction further the next section of this paper.

Using the Action Items devised by the meeting group, the first draft of the VAS attempted to summarise the problems with the system that we had heard and make recommendations for improvement. This draft was circulated back to AKE participants in the spring of 2021 and underwent extensive revisions led by AB. By this time, connections made through the meetings themselves had already sparked significant policy change prior to the completion of the VAS. For example, connections made between the City of Vancouver, EIDGE, and the Drinkers Lounge during AKE meetings surrounding the attempted removal of a bus stop on the 800 block East Hastings Street, where drinkers have historically congregated aided the opening of a harm reduction-focused parklet space at the Drinkers Lounge itself. The Drinkers Lounge parklet allowed a regular, predominately Indigenous, clientele to continue to access MAP program and cultural reconnections, while capacity limits were in place throughout the neighbourhood. A newly sanctioned space on the 800 block similarly provided illicit drinkers with access to picnic tables, a warming tent, bathrooms, and harm reduction services in partnership with a leading local non-profit geared towards providing services to sex workers [46]. In addition, a collaboration between Pivot Legal Society, EIDGE, and the Drinkers Lounge resulted in the creation of a “Drinkers Rights Card”, a wallet-sized information sheet with legal information that drinkers can use to protect themselves from police harassment and liquor pour outs [47]. The simple act of convening was more than enough to prompt these collaborations before the policy development process had even officially begun. We believe that this experience speaks to the untapped energy that surrounds intersectoral collaboration on alcohol harm reduction in the DTES, should such an initiative continue to be properly resourced.

## “We do our own policy”: Bringing illicit drinkers to the forefront of alcohol policy

Decades of community organizing and collaboration between people with lived and living experience of illicit substance use, academics, and public health researchers has resulted in the frequent involvement of peers drug policy-related research in B.C. Several drug user groups in the DTES, including VANDU and the now defunct SALOME–NAOMI Association of Patients (SNAP), and the Coalition of Peers Dismantling the Drug War (CPDDW) have routinely demanded a meaningful role for drug users in the ethical production of knowledge that is non-extractive and results in material benefits to the community [25, 48–52]. Accordingly, rich scholarship relating to the use of Participatory Action Research (PAR) in the Downtown Eastside has been produced in Vancouver [48, 49, 53–55]. PAR-informed research typically aims to empower and engage community members, traditional research “subjects”, in determining research questions through problem posing, identifying the structural determinants of adverse social conditions, carrying out data collection, reflexively interpreting findings, and translating results into concrete political advocacy [56, 57]. Importantly, the AKE project and production of the VAS were not conceived of as examples of PAR despite sharing several similarities, namely, a central role for peers in directing the recommendations and advising the political mobilization of the final document. We believe the AKE and VAS are more akin to participatory action *policy*, where illicit drinkers drew from years of experiential knowledge sharing to become directly involved in constructing alcohol-related policy in an informal organizing environment. The knowledge that informed the creation of the VAS had been developed and shared by a community of illicit drinkers living in the DTES for over a decade before being drawn upon to guide an action-oriented policy design process. EIDGE’s inclusion as a collective author for this commentary recognizes the importance of community knowledge to the nature of our work, which is difficult to attribute to individuals. Despite the close relationship between our work and the extensive participatory literature involving people who drugs, the lack of formal qualitative methods beyond regular organizing meetings and our reliance on a body of lived knowledge developed by EIDGE over years creates some distance between the AKE, VAS and PAR. The conditions and experiences of organizing at VANDU simply demanded a different, more pragmatic, and abridged approach to alcohol policy design that we describe in this section of our commentary.

**Fig. 3:**
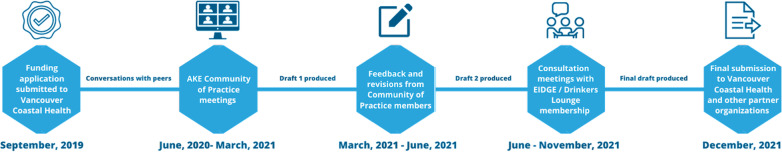
Timeline of the Alcohol Knowledge Exchange and Vancouver Alcohol Strategy development process

The remote nature of early AKE community of practice meetings and resulting achievements did not come without trade-offs. With the exception of 1 to two EIDGE members who could be supported to attend meetings alongside BG, COVID-19-related public health restrictions and the closure of neighbourhood services prevented illicit drinkers themselves from participating in this stage of the AKE project. While individual peers with lived and living experience of illicit alcohol use were included on calls whenever it was possible, a lack of reliable digital communication resources and social isolation prevented the vast majority of the EIDGE membership from contributing to the AKE discussions. Although AKE meetings had been initially structured around issues identified by EIDGE as pressing to the community, the earliest draft of the VAS did not directly reflect the direct input of people with lived and living experience of illicit drinking. It was imperative that further drafts of the VAS be collaboratively reviewed and changed by illicit drinkers themselves as soon as it was feasible to ensure that eventual policy recommendations came from and had the endorsement of the community. A full timeline of the AKE and VAS development process is depicted in Fig. 3.

The easing of COVID-19 case counts in the DTES in the early summer of 2021 allowed us to convene a series of peer consultation meetings to this end. We decided to create a joint-organizational VAS reading group between the membership and EIDGE and the Drinkers Lounge that would meet to discuss working drafts of each section of the document as they were written. We hosted 8 of these meetings between June and November 2021. For a 6-month period, interested peers from each group met in Oppenheimer Park, the geographic and social heart of the DTES. The Park itself is located directly between the office of the Vancouver Area Network of Drug Users and the Drinkers Lounge, and drinkers' pre-existing feeling of safety and community in the park made it a natural choice of venue. We printed copies of the strategy, ordered coffee, and offered members $10 stipends, $5 more than the usual meeting stipend offered by VANDU, to attend these biweekly meetings which lasted for 1 h. Week by week, a group of between 15 and 30 participants read and discussed each of the 6 thematic areas for policy change that has emerged from the AKE community of practice, making corrections and additions to better reflect drinkers’ priorities and changing conditions in the DTES related to amenity access, policing, and more. At each meeting, AB facilitated discussion and took detailed notes on participants feedback. Then, AB incorporated said revisions into the recommendations of the VAS and returned new drafts to the biweekly meeting for discussion and approval. Existing recommendations were also reordered by AB following discussion by the group to better reflect drinkers needs as priority policy problems became clear. In one instance, access to safe and clean washrooms for illicit drinkers was repeatedly raised as an urgent area for policy action that impacted the daily lives of people who drank outside. The VAS’ discussion of washrooms soon became longer, more detailed, and more prominently featured in the final document.

These meetings were a critical piece of the strategy’s development process and justify our framing of the VAS as a grassroots policy document. Illicit drinkers were not being confined to the margins or superficially “consulted” in a tokenized manner. The original foci of the AKE project were drawn from discussions with drinkers, so it was, therefore, appropriate that drinkers have the final say over the demands of the alcohol strategy that was designed to respond to them. The VAS appears to be novel in this regard, as we are not aware of a similar alcohol policy guideline document existing elsewhere in Canada that has been written to centre the perspectives of illicit drinkers. This too is an outcome of drinkers' intense structural marginalization, their exclusion from harm reduction organizing, and the systemic under-resourcing of alcohol-specific harm reduction programming throughout Canada. Once the venue for this form of consultation was made available, drinker’s enthusiasm and engagement was consistent. To let members know about upcoming meetings, we made announcements at weekly EIDGE meetings, connected with drinkers one-on-one in the neighbourhood, and distributed posters at VANDU and the PHS Drinkers Lounge. Peers who used alcohol were excited about the opportunity to discuss alcohol policy. When given the opportunity to do so, illicit drinkers are ready and willing to discuss alcohol harm reduction and policy reform. Contrary to stereotypes of illicit drinkers as apathetic or uninformed, the degree to which alcohol policy “touches down” in the DTES, oftentimes creating harm, was readily apparent to attendees. When the meetings drew to a close in preparation for the writing of the final Vancouver Alcohol Strategy, the group expressed disappointment. There was an untapped energy between illicit drinkers to continue developing public health policy, and we committed to continuing the Wednesday Oppenheimer Park meetings should resources become available to do so. Our regular meetings at the picnic benches at Oppenheimer Park had been a model for alcohol policy development of a different sort. No longer content to remain an unconsidered exception to population-level alcohol policies and public health initiatives, illicit drinkers living in the DTES enthusiastically gathered to share stories and offer feedback on a framework for their own future.

## The recommendations of the Vancouver Alcohol Strategy

MH reviewed several versions of the VAS document before the final version of the VAS document was finalized in December of 2021. The strategy was presented to Vancouver Coastal Health’s Substance Use and Priority Populations team in January of 2022. This final version included 47 unique alcohol policy recommendations across 6 thematic areas for action, derived from early conversations of the AKE community of practice and refined through peer consultations. Since then, AB, BG, MH, and GS have presented the process for creating the VAS and it’s recommendations at webinars and academic conferences in B.C. In the section that follows, we provide a brief synopsis of each thematic area for policy change and include an example recommendation for each. The VAS document is organized thematically rather than by jurisdiction, meaning that individual policy recommendations pertaining to multiple levels of government but sharing some commonality are often included alongside one another. As an advocacy tool, the VAS is designed to guide policymakers and community members towards a better understanding of the driving forces behind illicit drinkers structural marginalization and policy domains that impact illicit drinkers. That said, the VAS is intended to inspire but not necessarily guide advocacy on an issue-by-issue basis, summarizing concerns without offering detailed legislative plans of action for each level of government that is implicated. A sample of recommendations included in the VAS are as follows:

### Action item 1: equity-focused decriminalization of drinkers

People who drink in public spaces and people who use non-beverage alcohol in Vancouver continue to be targeted, harassed, and criminalized by law enforcement. The Downtown Eastside in particular is a hyper-surveyed and over-policed environment, where Indigenous drinkers are particularly at risk of police interactions. Drinkers who encounter police are frequently subjected to liquor pour outs, ticketing, and the possibility of temporary incarceration, all of which have serious consequences for health and well-being of low-income persons who are dependent on alcohol. The absence of a dedicated sobering centre in the neighbourhood means that intoxicated drinkers who are taken into custody are usually placed in a holding cell, where they are at serious risk of assault and injury associated with inadequate care. Accordingly, the VAS calls for the following:

- 1.1 Suspend the enforcement of all provincial statutes, local bylaws and park regulations related to the use of alcohol in public spaces throughout the Downtown Eastside that criminalize illicit drinkers and further marginalise precariously housed residents who use alcohol.
- 1.3 Establishing alternative pathways to the criminal justice system for illicit drinkers in Vancouver (Including safe ride programs and a civilian run sobering centre).

### Action item 2: creating safe indoor and outdoor spaces for drinkers

We repeatedly heard illicit drinkers discuss the importance of safe indoor spaces that accommodate people who primarily use alcohol. The group was also acutely aware of the absence of public amenities such as benches, parklets, plazas, washrooms, covered sitting areas, bus stops, and bus stop rain shelters throughout the neighbourhood and its adjacent parks. The need for outdoor spaces for illicit drinkers to congregate is compounded by the presence of exclusionary policies and practices within community drop-in spaces, harm reduction programming sites and shelters, where staff are not typically trained to meet drinkers’ health and behavioural needs or equipped with alcohol-specific policies to guide them. To the peers who were involved with the writing of this strategy, these conditions reflect the broader neglect of the DTES by city planners and governmental officials and result in threats to drinkers safety. Therefore, we called for the following in the VAS:

- 2.2 Financial and logistical support for existing community spaces to accommodate drinkers needs.
- 2.3 Continued Support for Drinker-Friendly Parks and Parklets.
- 2.4 Improved Access to Outdoor Amenities.
- 2.5 Improved Access to Water, Hygiene and Sanitation Infrastructure.
- 2.6 Creating a Network of Peer-Led, Safe Warming Sites and Sobering Centres in Downtown Vancouver.

### Action item 3: managed alcohol programs and safe housing

Attendees of our peer consultation meetings saw adequately resourced and properly run Managed Alcohol Programs (MAPs) as a central part of the ideal standard of care for themselves and many of their peers. MAPs provided participants with a safer and consistent supply of beverage alcohol, nutritious food, contact with primary care providers, social connection and acceptance, cultural reconnection, referral to housing supports and other services, and consistent employment. These views were consistent with the published literature supporting MAP efficacy [58–66]. The community-led brewing cooperative model implemented by the Drinkers Lounge, one that involves peers in the administration of the site as well as its wraparound programming, was touted as an example for other organizations to follow throughout Vancouver [63]. Illicit drinkers expressed a desire for more MAPs, better resourcing for existing ones, and a diversification of MAP models to include off-site delivery and outreach-based MAP. Organizers continued to express frustration that more MAPs had not been opened around the DTES, even as high quality evidence from the Canadian Managed Alcohol Programs Study (CMAPS) was published. Illicit drinkers also spoke to the importance of safe and accommodating housing for reducing alcohol-related harms. Specifically, participants called for an end to “no-guest” policies in SROs, expanded supports in supportive housing that do not undermine tenancy rights, expanded home-detox services, improved staff training and outreach-based? In privately owned buildings. The VAS reflects these discussions by recommending:

- 3.2 More dedicated Managed Alcohol Programs in and around the DTES in accordance with Vancouver Coastal Health’s DTES Second Generation Strategy.
- 3.3 Increased resources for Vancouver’s existing Managed Alcohol Programs and alcohol-specific harm reduction organisations, including COVID-19 MAPs, all of which are attempting to accommodate increased demand for services despite being pushed beyond their capacity by COVID-19.
- 3.6 VCH-produced guidance materials for private for-profit and non-profit housing providers to design, develop and implement MAPs in collaboration with VCH, drinkers and local service providing organizations.

### Action item 4: expanding and reforming addiction treatment services throughout metro-Vancouver

The VAS focus on harm reduction includes an enthusiastic acknowledgement of the critical role that detox and in-patient treatment programs have to play in meeting drinkers' needs along a continuum of use. Many participants in the peer consultations that informed the writing of this strategy had prior experience with treatment and recovery services, and were willing to share their experiences in hopes of improving the systems responsiveness. Illicit drinkers recognized the need for publicly funded detox beds on demand, access to appropriate medications for the management of withdrawal, and the rescinding of smoking bans in detox centres operated by VCH. The priority recommendations of our membership were as follows:

- 4.5 Reforms to residential detox and treatment centres rules and regulations, namely, the elimination of smoking bans at detox centers and the. Creation of designated smoking areas wh.
- 4.1 Reducing wait times and increasing the availability of beds and outreach services.
- 4.3 Establishing a durable and properly funded interdisciplinary network of post-detox follow-up, referral, system navigation and support coordinated by VCH, the Ministries of Health and Mental Health and Addictions, and local service providers.

### Action item 5: peer-led education for clients and practitioners

The impetus of the VAS itself was creating a venue, where the underappreciated knowledge and experiences of illicit drinkers themselves could contribute to reshaping Vancouver’s system of alcohol treatment, harm reduction, and related care. EIDGE members have been pushing for reforms to the alcohol harm reduction sector for over a decade and have been integral to creating several advancements in alcohol research and care including consulting on the British Columbia Centre on Substance Use’s Guidelines for the Clinical Management of High-Risk Drinking and Alcohol Use Disorder and the Operational Guidance for Implementation of Managed Alcohol for Vulnerable Populations. Yet, when we have asked for specific policy changes like increased funding supports for Managed Alcohol Programs or removal of the no smoking ban at detox facilities, our perspectives and requests have been largely ignored. At the moment, there is no single place to go to access up to date alcohol-related public health and programming information in Vancouver, and service providers are extremely disconnected from the realities of long-term, heavy alcohol use in the DTES. Illicit drinkers, activists, and service providers believed that a fundamental restructuring of how services were connected with one another should be included within the VAS, and that drinkers should have a leading role to play in educating members of the AKE network going forward. We subsequently recommended the following:

- 5.1 The development of a centralised online platform for stakeholders working in the area of alcohol policy and harm reduction that operates through the VCH website.
- 5.2 Accessible and publicly available resources for clients and service providers designed by peers.
- 5.7 Evidence-based and peer-directed Harm Reduction Education for health care Practitioners and social service providers.

Everyone involved with the AKE project and the convening of the joint-organisational meetings in Oppenheimer Park recognized that the very act of connecting was generative. Coming together in an organized way, with the proper resources to do so, could continue to create policy change related to alcohol harm reduction. We, therefore, emphasised the following to Vancouver Coastal Health:

- 5.3 Provide financial support for regular meetings of the newly formed AKE Community of Practice network of service providers to maintain the system’s responsiveness and continue effective knowledge translation activities.
- 5.4 Provide financial support for regular joint meetings of the EIDGE and Drinkers Lounge membership to advise the newly formed AKE Community of Practice network of service providers to maintain the system’s responsiveness and continue effective knowledge translation activities.

### Action item 6: establishing long-term partnerships with governmental partners

The final recommendations of the VAS acknowledged that many of the policy changes we had agreed to demand were the responsibility of different levels of government. Although the strategy itself was prepared for the local health authority alone, we believed that it was important to include a section detailing future partnerships, because VCH was well-positioned to work with our membership as a political ally to continue to advocate for recommendations that fell beyond its jurisdiction. Examples of these recommendations include:

- 6.2 Assist B.C. Housing to develop and implement MAPs where appropriate and supported by the Ministries of the Attorney General, Health, and Mental Health and Addictions as design as well as implement tenant-suggested best practices for alcohol harm reduction in B.C. Housing facilities.
- 6.7 The creation of MAP-specific licensing requirements for the distribution of alcohol from the Liquor Distribution Branch and Liquor and Cannabis Regulation Branch of the Ministry of the Attorney General that enable the development of new programs unhindered by concerns surrounding their legality.
- 6.11 Requiring an equity-focused policy impact assessment and response plan for changes to alcohol pricing that occur at the provincial level, including changes to minimum unit prices and alcohol taxation.
- 6.12 Develop enforceable standards of practice for interacting with drinkers for law enforcement officers in Vancouver and throughout the province. These guidelines should direct officers away from punitive enforcement measures, including fines, charges, liquor pour-outs and confiscation when interacting with drinkers in Vancouver and emphasise the negative health consequences associated with said measures.

## Drinker-led organizing for public health policy implementation

The VAS is a significant achievement in Vancouver’s drug policy history that attempts to address the historical exclusion of illicit drinkers from an increasingly formalised and apolitical harm reduction sphere. The militant spirit of the movement continues to be kept alive by organisations, such as VANDU, and it was, therefore, appropriate that illicit drinkers affiliated with EIDGE lead a process of drinker-involved policy development in the tradition of “nothing about us without us”. The creation of alcohol policy in Canada has historically been largely led by highly professionalized experts, trained public health professionals, and epidemiologists. The opinions and experience of illicit drinkers themselves, who experience the impacts of B.C.’s system of alcohol regulation in a unique way on a daily basis, have not been consistently integrated into population-level or harm reduction-informed alcohol policy**.** The process of policy development undertaken by the VAS has attempted to unmake and resist this approach to public health policy development related to alcohol control by intentionally creating space for people with lived experience to guide our recommendations. This would not be possible without the networks of trust and solidarity cultivated through decades of drug user activism housed within VANDU, and active work to connect drinkers throughout the neighbourhood by peers and staff members. Historical reflections have demonstrated to us and our membership that the tacit and overt exclusion of drinkers in the creation of alcohol policies for Vancouver’s Downtown Eastside has contributed to alcohol-related harm for acutely marginalized drinkers in the area [42]. By respecting lived knowledge and centring it within a wider project of Alcohol Knowledge Exchange, we believe that we are well-positioned to continue working to redress the structurally rooted vulnerability of our friends, peers, mentors, and colleagues.

We believe that our experience organising the AKE project and producing the VAS alongside people who use illicit alcohol provides several key lessons for public health and social service professionals seeking to do truly community-driven alcohol policy work building on the principles of PAR. To begin, we urge public health practitioners to ground their research and advocacy work in meaningful partnership with organisations run by and for people who use drugs. Doing so ensures that the most pressing and immediate issues facing peers who use alcohol are the focus around which policy solutions develop. Prior to convening our community of practice, we ensured that our conversations would clearly address topics that EIDGE group had identified as significant barriers to accessing adequate alcohol-related care and support in the DTES. We cannot overstate the degree to which the lived experience of illicit drinkers informed the nature and order of the recommendations written into the VAS by AB, BG, and peer reviewers including MH. Drinkers are intimately familiar with the ways in which the present system of alcohol harm reduction, detox, treatment, and clinical care pushes peers away. They possess a degree of knowledge about the causes and potential solutions of alcohol-related harm in the DTES that is unrivalled and often draws from firsthand experience. Illicit drinkers have spent years navigating the neighbourhood's selectively prohibitionist retail alcohol landscape, taking care of their peers to prevent dehydration and hypothermia, advocating to health care professionals, and struggling to open new MAPs. Necessity and immense loss have led to the development of peer support systems outside of any formalised program, and given drinkers insight into potentially impactful interventions that are beyond the traditional purview of public health. Illicit drinkers' emphasis on the installation and preservation of public amenities in the DTES is an effective case in point—to peers, alcohol harm reduction is inseparable from the broader struggle to remain in place within a neighbourhood that has excluded them from its planning and politics for decades. We are hopeful that the recommendations of the VAS and our description of its development process can support communities of drinkers elsewhere to organize towards alcohol policy equity while providing health-service professionals and policymakers with a replicable roadmap for doing meaningfully community-led work where they are.

## Data Availability

Not applicable.

## References

[1] British Columbia Coroners Service*. *Illicit Drug Toxicity Deaths in BC, January 1, 2012–June 30, 2022*.* 2022 Aug. https://www2.gov.bc.ca/assets/gov/birth-adoption-death-marriage-and-divorce/deaths/coroners-service/statistical/illicit-drug.pdf

[2] Vancouver Coastal Health. CMHO Report 2018: Response to the Opioid Overdose Crisis in Vancouver Coastal Health. 2018. http://www.vch.ca/Documents/CMHO-report.pdf

[3] Crabtree A. It’s powerful to gather: a community-driven study of drug users' and illicit drinkers' priorities for harm reduction and health promotion in British Columbia, Canada (Doctoral dissertation, University of British Columbia).

[4] Crabtree A, Latham N, Bird L, Buxton J (2016). Results of a participatory needs assessment demonstrate an opportunity to involve people who use alcohol in drug user activism and harm reduction. Harm Reduct J.

[5] Crabtree A, Latham N, Morgan R, Pauly B, Bungay V, Buxton JA (2018). Perceived harms and harm reduction strategies among people who drink non-beverage alcohol: community-based qualitative research in Vancouver, Canada. Int J Drug Policy.

[6] Brown L, Skulsh J, Morgan R, Kuehlke R, Graham B (2018). Research into action? The Eastside Illicit Drinkers Group for Education's (EIDGE) experiences as a community-based group in Vancouver, Canada. Drug Alcohol Rev.

[7] Kesselring S (2013). Illicit alcohol in British Columbia.

[8] The Eastside Illicit Drinkers Group for Education. Understanding EIDGE: illicit alcohol use in the DTES. 2017.

[9] Hasson S, Ley D (1994). Neighbourhood organizations and the welfare state.

[10] Campbell L, Boyd N, Culbert L (2009). A thousand dreams: Vancouver's Downtown Eastside and the fight for its future.

[11] Masuda J (2023). Right to remain research collective. Abandoning the SRO: public health withdrawal from sanitary enforcement in Vancouver’s Downtown Eastside. J Urban History.

[12] Blomley N (2021). Right to remain collective. Making property outlaws: law and relegation. Int J Urban Reg Res.

[13] Fleming T, Damon W, Collins AB, Czechaczek S, Boyd J, McNeil R (2019). Housing in crisis: a qualitative study of the socio-legal contexts of residential evictions in Vancouver’s Downtown Eastside. Int J Drug Policy.

[14] Anderson KJ (1987). The idea of Chinatown: the power of place and institutional practice in the making of a racial category. Ann Assoc Am Geogr.

[15] Anderson KJ. Vancouver's Chinatown: racial discourse in Canada, 1875–1980. McGill–Queen's Press-MQUP, Montreal; 1991

[16] Wideman TJ, Masuda JR (2018). Assembling “Japantown”? A critical toponymy of urban dispossession in Vancouver. Can Urban Geogr.

[17] Masuda JR, Franks A, Kobayashi A, Wideman T (2020). After dispossession: an urban rights praxis of remaining in Vancouver’s Downtown Eastside. Environ Plan D: Soc Space.

[18] Bailey A. Historicizing Vancouver’s Liquor License Moratorium for the Downtown Eastside as Dispossessory Public Health Practice. Masters thesis, Queen's University (Canada).

[19] Boyd SC, Osborn B, MacPherson D. Raise shit!: social action saving lives. Fernwood; 2009.

[20] Lupick T (2018). Fighting for space: how a group of drug users transformed one city’s struggle with addiction.

[21] Francis D. Red light neon: a history of Vancouver's sex trade. Subway; 2006.

[22] Ross BL (2010). Sex and (evacuation from) the city: the moral and legal regulation of sex workers in Vancouver’s West End, 1975–1985. Sexualities.

[23] Jozaghi E (2014). The role of drug users’ advocacy group in changing the dynamics of life in the Downtown Eastside of Vancouver. Can J Subst Use.

[24] Jozaghi E (2015). Exploring the role of an unsanctioned, supervised peer driven injection facility in reducing HIV and hepatitis C infections in people that require assistance during injection. Health Justice.

[25] Jozaghi E, Greer AM, Lampkin H, Buxton JA (2018). Activism and scientific research: 20 years of community action by the Vancouver area network of drug users. Subst Abuse Treat Prev Policy.

[26] Kerr T, Mitra S, Kennedy MC, McNeil R (2017). Supervised injection facilities in Canada: past, present, and future. Harm Reduct J.

[27] Bardwell G, Fleming T, Collins AB, Boyd J, McNeil R (2019). Addressing intersecting housing and overdose crises in Vancouver, Canada: opportunities and challenges from a tenant-led overdose response intervention in single room occupancy hotels. J Urban Health.

[28] Nowell M, Masuda JR (2020). “You need to just provide health services:” navigating the politics of harm reduction in the twin housing and overdose crises in Vancouver. BC Int J Drug Policy.

[29] York F. The Highs and Lows of SROs: Rents and the rate of change in the Downtown Eastside: 2019–2020 COVID-Eye’zd CCAP hotel survey & housing report.

[30] Weaver PK. The regionalization of health care in British Columbia: does’ closer to home’really matter?

[31] Masuda JR, Robinson K, Elliott SJ, Eyles J (2012). Chronic disease prevention and the politics of scale: lessons from Canadian health reform. Soc Work Public Health.

[32] Hasenfeld Y, Garrow EE (2012). Nonprofit human-service organizations, social rights, and advocacy in a neoliberal welfare state. Soci Serv Rev.

[33] Vancouver Coastal Health. Downtown Eastside Second Generation Health System Strategy. [Internet]. Vancouver (B.C.). 2015. Available from: https://www.vch.ca/sites/default/files/2022-11/DTES-Second-Generation-Health-System-Strategy-Design-Paper.pdf

[34] Masuda JR, Chan S (2016). Vancouver coastal health’s second generation health strategy: a need for a reboot?. Can J Public Health.

[35] Butt P, Beirness D, Gliksman L, Paradis C, Stockwell T (2011). Alcohol and health in Canada: a summary of evidence and guidelines for low-risk drinking.

[36] Stockwell T, Zhao J, Macdonald S, Vallance K, Gruenewald P, Ponicki W, Holder H, Treno A (2011). Impact on alcohol-related mortality of a rapid rise in the density of private liquor outlets in British Columbia: a local area multi-level analysis. Addiction.

[37] Stockwell T, Auld MC, Zhao J, Martin G (2012). Does minimum pricing reduce alcohol consumption? The experience of a Canadian province. Addiction.

[38] Treno AJ, Ponicki WR, Stockwell T, Macdonald S, Gruenewald PJ, Zhao J, Martin G, Greer A (2013). Alcohol outlet densities and alcohol price: The British Columbia experiment in the partial privatization of alcohol sales off-premise. Alcohol Clin Exp Res.

[39] Thompson K, Stockwell T, Wettlaufer A, Giesbrecht N, Thomas G (2017). Minimum alcohol pricing policies in practice: a critical examination of implementation in Canada. J Public Health Policy.

[40] Campbell CA, Hahn RA, Elder R, Brewer R, Chattopadhyay S, Fielding J, Naimi TS, Toomey T, Lawrence B, Middleton JC (2009). Task force on community preventive services. The effectiveness of limiting alcohol outlet density as a means of reducing excessive alcohol consumption and alcohol-related harms. Am J Prev Med.

[41] Livingston M (2011). Alcohol outlet density and harm: comparing the impacts on violence and chronic harms. Drug Alcohol Rev.

[42] Bailey A., the Eastside Illicit Drinkers Group for Education. Alcohol prohibition never ended in Vancouver’s Downtown Eastside. We see another way forward. The Mainlander. 2021.

[43] Wilt J (2022). Drinking up the revolution: how to smash big alcohol and reclaim working class joy.

[44] Westenberg JN, Kamel MM, Addorisio S, Abusamak M, Wong JS, Outadi A, Jang KL, Krausz RM (2021). Non-beverage alcohol consumption among individuals experiencing chronic homelessness in Edmonton, Canada: a cross-sectional study. Harm Reduct J.

[45] Jesse S, Bråthen G, Ferrara M, Keindl M, Ben-Menachem E, Tanasescu R (2017). Alcohol withdrawal syndrome: mechanisms, manifestations, and management. Acta Neurol Scand.

[46] Fumano D. “Public drinking as a health measure—Vancouver eyes allowing booze in DTES ‘parklet,” The Vancouver Sun (February 22nd, 2022). https://vancouversun.com/news/local-news/dan-fumano-public-drinking-as-a-health-measure-vancouver-eyes-allowing-booze-in-dtes-parklet

[47] https://www.pivotlegal.org/rights_card_alcohol_seizure

[48] Boilevin L, Chapman J, Deane L, Fresz G, Joe DJ, Leech-Crier N, Winter P. A manifesto for ethical research in the Downtown Eastside.

[49] Neufeld SD, Chapman J, Crier N, Marsh S, McLeod J, Deane LA (2019). Research 101: a process for developing local guidelines for ethical research in heavily researched communities. Harm Reduct J.

[50] Boyd S, NAOMI Patients Association (2013). Yet they failed to do so: Recommendations based on the experiences of NAOMI research survivors and a call for action. Harm Reduct J.

[51] Boyd S, Murray D, MacPherson D (2017). Telling our stories: heroin-assisted treatment and SNAP activism in the Downtown Eastside of Vancouver. Harm Reduct J.

[52] Boyd SU, Murray DA. Ethics, research, and advocacy: The experiences of the NAOMI patients association in Vancouver’s Downtown Eastside. Crit Inq Soc Just Mental Health. 2017:365–85.

[53] Boyd SC (2008). Community-based research in the Downtown Eastside of Vancouver. Resour Fem Res.

[54] Damon W, Callon C, Wiebe L, Small W, Kerr T, McNeil R (2017). Community-based participatory research in a heavily researched inner city neighbourhood: perspectives of people who use drugs on their experiences as peer researchers. Soc Sci Med.

[55] Masuda J, Kobayashi A. Participatory research by/for the precariously housed in a time of COVID-19. InCOVID-19 and Similar futures: pandemic geographies 2021 (pp. 357–363). Cham: Springer International Publishing.

[56] Baum F, MacDougall C, Smith D (2006). Participatory action research. J Epidemiol Community Health.

[57] Baum FE (2016). Power and glory: applying participatory action research in public health. Gac Sanit.

[58] Vallance K, Stockwell T, Pauly B, Chow C, Gray E, Krysowaty B (2016). Do managed alcohol programs change patterns of alcohol consumption and reduce related harm? A pilot study. Harm Reduct J.

[59] Stockwell T, Pauly B, Chow C, Erickson RA, Krysowaty B, Roemer A (2018). Does managing the consumption of people with severe alcohol dependence reduce harm? A comparison of participants in six Canadian managed alcohol programs with locally recruited controls. Drug Alcohol Rev.

[60] Stockwell T, Pauly B (2018). Managed alcohol programs: is it time for a more radical approach to reduce harms for people experiencing homelessness and alcohol use disorders?. Drug Alcohol Rev.

[61] Ivsins A, Pauly B, Brown M, Evans J, Gray E, Schiff R et al. On the outside looking in: finding a place for managed alcohol programs in the harm reduction movement. Int J Drug Policy. 2019;67: 58–62. 10.1016/j.drugpo.2019.02.004 30959410

[62] Pauly B, Brown M, Evans J, Gray E, Schiff R, Ivsins A (2019). “There is a Place”: impacts of managed alcohol programs for people experiencing severe alcohol dependence and homelessness. Harm Reduct J.

[63] Pauly B, King V, Smith A, Tranquilli-Doherty S, Wishart M, Vallance K (2021). Breaking the cycle of survival drinking: insights from a non-residential, peer-initiated and peer-run managed alcohol program. Drugs Educ Prev Policy.

[64] Stockwell T, Zhao J, Pauly B, Chow C, Vallance K, Wettlaufer A (2021). Trajectories of Alcohol use and related harms for managed alcohol program participants over 12 months compared with local controls: a quasi-experimental study. Alcohol Alcohol.

[65] Evans J, Semogas D, Smalley JG, Lohfeld L (2015). “This place has given me a reason to care”: understanding ‘managed alcohol programs’ as enabling places in Canada. Health Place.

[66] Carver H, Parkes T, Browne T, Matheson C, Pauly B (2021). Investigating the need for alcohol harm reduction and managed alcohol programs for people experiencing homelessness and alcohol use disorders in Scotland. Drug Alcohol Rev.

